# Higher docosahexaenoic acid levels lower the protective impact of eicosapentaenoic acid on long-term major cardiovascular events

**DOI:** 10.3389/fcvm.2023.1229130

**Published:** 2023-08-23

**Authors:** Viet T. Le, Stacey Knight, Jeramie D. Watrous, Mahan Najhawan, Khoi Dao, Raymond O. McCubrey, Tami L. Bair, Benjamin D. Horne, Heidi T. May, Joseph B. Muhlestein, John R. Nelson, John F. Carlquist, Kirk U. Knowlton, Mohit Jain, Jeffrey L. Anderson

**Affiliations:** ^1^ Intermountain Medical Center, Intermountain Heart Institute, Salt Lake City, UT, United States; ^2^ Department of Physician Assistant Studies, Rocky Mountain University of Health Professions, Provo, UT, United States; ^3^ The University of Utah, School of Medicine, Salt Lake City, UT, United States; ^4^ Department of Medicine, University of California San Diego, San Diego, CA, United States; ^5^ Division of Cardiovascular Medicine, Department of Medicine, Stanford University, Stanford, CA, United States; ^6^ California Cardiovascular Institute, Fresno, CA, United States

**Keywords:** EPA, DHA, major adverse cardiovascular events, omega-3, outcomes

## Abstract

**Introduction:**

Long-chain omega-3 polyunsaturated fatty acids (OM3 PUFA) are commonly used for cardiovascular disease prevention. High-dose eicosapentaenoic acid (EPA) is reported to reduce major adverse cardiovascular events (MACE); however, a combined EPA and docosahexaenoic acid (DHA) supplementation has not been proven to do so. This study aimed to evaluate the potential interaction between EPA and DHA levels on long-term MACE.

**Methods:**

We studied a cohort of 987 randomly selected subjects enrolled in the INSPIRE biobank registry who underwent coronary angiography. We used rapid throughput liquid chromatography-mass spectrometry to quantify the EPA and DHA plasma levels and examined their impact unadjusted, adjusted for one another, and fully adjusted for comorbidities, EPA + DHA, and the EPA/DHA ratio on long-term (10-year) MACE (all-cause death, myocardial infarction, stroke, heart failure hospitalization).

**Results:**

The average subject age was 61.5 ± 12.2 years, 57% were male, 41% were obese, 42% had severe coronary artery disease (CAD), and 311 (31.5%) had a MACE. The 10-year MACE unadjusted hazard ratio (HR) for the highest (fourth) vs. lowest (first) quartile (Q) of EPA was HR = 0.48 (95% CI: 0.35, 0.67). The adjustment for DHA changed the HR to 0.30 (CI: 0.19, 0.49), and an additional adjustment for baseline differences changed the HR to 0.36 (CI: 0.22, 0.58). Conversely, unadjusted DHA did not significantly predict MACE, but adjustment for EPA resulted in a 1.81-fold higher risk of MACE (CI: 1.14, 2.90) for Q4 vs. Q1. However, after the adjustment for baseline differences, the risk of MACE was not significant for DHA (HR = 1.37; CI: 0.85, 2.20). An EPA/DHA ratio ≥1 resulted in a lower rate of 10-year MACE outcomes (27% vs. 37%, adjusted *p*-value = 0.013).

**Conclusions:**

Higher levels of EPA, but not DHA, are associated with a lower risk of MACE. When combined with EPA, higher DHA blunts the benefit of EPA and is associated with a higher risk of MACE in the presence of low EPA. These findings can help explain the discrepant results of EPA-only and EPA/DHA mixed clinical supplementation trials.

## Introduction

The American Heart Association (AHA) dietary guidelines have recommended that individuals at higher risk for cardiovascular disease (CVD) should consume at least two servings of fish per week or other food sources rich in long-chain omega-3 polyunsaturated fatty acid (OM3 PUFA) to achieve cardioprotective effects (1, 2). However, the efficacy of OM3 PUFA supplements remains controversial (3–6). The three main OM3 PUFAs recommended by the AHA are eicosapentaenoic acid (EPA), docosahexaenoic acid (DHA), and alpha-linolenic acid (ALA), which are commonly referred to as omega-3s (OM3s). While it has been a common cardiology practice to discuss OM3s with patients and recommend that they add or supplement their diets with OM3-containing food sources, several meta-analyses in the past decade have reported a lack of efficacy of OM3 supplements in reducing major adverse cardiovascular event (MACE) outcomes (3–7). Subsequently, in 2017, the AHA modified their recommendation to exclusively use OM3s in patients “with prevalent clinical CHD such as a recent myocardial infarction (MI)” (8). Two separate meta-analyses, one by Harris et al. (9), which evaluated the EPA and DHA circulating levels in 17 prospective cohorts on total and cause-specific mortality, and the other by Hu et al (10), which assessed the OM3 (EPA-only and EPA/DHA mixed) supplementation on cardiovascular disease risk reduction in 13 randomized, placebo-controlled trials (RCTs), demonstrated inverse relationships between both supplementation dose and EPA and DHA circulating levels and mortality and cardiovascular events. Furthermore, five contemporary RCTs reported markedly different outcomes. The 2018 REDUCE-IT (11) trial (EPA-only) achieved a 25% relative risk reduction (*p* < 0.00000001) in the composite MACE primary endpoint, whereas all four mixed OM3 (EPA and DHA) RCTs, namely, ASCEND (12), VITAL (13) (2018), STRENGTH (14), and OMEMI (15) (2020), found no association. Hu et al.’s (10) meta-analysis included two of the aforementioned RCT cardiovascular outcome trials (CVOTs), ASCEND and VITAL. Both used the same EPA/DHA mixed formulation and did not meet their primary endpoints.

Additionally, despite Hu et al.’s meta-analysis of OM3 supplementation RCT CVOTs which supported the benefit of EPA-only and EPA/DHA mixed formulation supplementation, the primary EPA-only (REDUCE-IT, JELIS) and EPA/DHA RCT CVOTs demonstrated different outcomes. Although Harris et al. demonstrated inverse relationships between OM3 circulating levels and mortality and cardiovascular events, they did not test for interactions between various OM3 PUFA biomarkers. We hypothesized that DHA might interact with the MACE risk-reducing (all-cause death, MI, stroke, heart failure hospitalization 16–18) effects of EPA. The Intermountain Healthcare subjects enrolled in the INSPIRE biobank registry (formerly known as the Intermountain Heart Collaborative Study) (19) were examined for the impact of EPA and DHA circulating levels unadjusted, adjusted for one another, and fully adjusted for comorbidities, EPA + DHA, and the EPA/DHA ratio on long-term (10-year) MACE.

## Materials and methods

### Study aim

This observational study aimed to evaluate whether plasma levels of EPA and DHA collected at baseline coronary angiography are associated with lower MACE on a 10-year follow-up. This study was approved by the Intermountain Healthcare Institutional Review Board with a waiver of consent and was conducted in compliance with the Declaration of Helsinki. This research was funded by an unrestricted grant that was provided through the philanthropy of the Dell Loy Hansen Heart Foundation.

### Study population

From the INSPIRE biobank registry, we randomly selected a cohort of 1,000 unique subjects who underwent their first coronary angiography at Intermountain Healthcare from January 1, 1994, to December 31, 2012. The cohort sample size was chosen to allow for a power of 80% with a baseline event rate of 0.25 and hazard ratio of 0.70 (α = 0.01). The INSPIRE biobank registry began in 1993 (19) and had a total of 19,000 unique subjects enrolled from 1994 to 2012. Being part of the INSPIRE biobank registry, subjects consented to have a blood sample taken at the time of their angiography. These samples included four EDTA tubes of blood that were processed for the separation of plasma and leukocyte DNA within 1 h of collection. The plasma was then transferred to RNase/DNase-free tubes and stored in temperature-controlled freezers at −80°C.

### Metabolite analysis

Plasma metabolites were tested at the University of San Diego's metabolomics laboratory (MJ, MN, KD, JW) by rapid throughput liquid chromatography-mass spectrometry and were reported in arbitrary spectral units (ASU) (20, 21). The results of the mass spectrometry underwent standard quality checking to eliminate metabolites and low-quality samples (20). All analyses were performed for internal consistency using ASU. The coefficient of variation, based on ASU, was 27.2 for EPA and 46.1 for DHA. Using absolute concentration values for the 100 randomly selected samples, we estimated a mean EPA concentration (mcg/ml) of 20.42 ± 10.17 mcg/ml and mean DHA concentration of 52.14 ± 17.58 mcg/ml (see Supplementary File for details).

### Clinical and outcomes data

Intermountain Health is an integrated, not-for-profit healthcare system consisting of 24 hospitals and 215 clinics providing approximately 65% of medical care in Utah and parts of Idaho and Nevada. Intermountain Health uses one electronic medical record (EMR) system consolidated into a single electronic data warehouse, which allows the capturing and tracking of patient outcomes across outpatient, emergency, and inpatient visits. The outcomes from the EMR are supplemented with death certificate data from the Utah Department of Health and the Social Security Death Master Index. Patient demographics and clinical characteristics prior to angiography, angiographic results, and testing results were obtained from the EMR. The primary endpoint for the study was a 10-year MACE, defined either as all-cause death, MI after the first 60-day post-angiography, stroke, or heart failure hospitalization. Clinical endpoints (e.g., MI, stroke, and heart failure) were determined using diagnostic codes and laboratory values (e.g., troponins for MI). For those with less than 10 years of follow-up, their outcomes were tracked until September 2020. Comorbidities were determined prior to or at the time of the baseline angiography. Severe coronary artery disease (CAD) was defined as ≥70% narrowing in a major coronary artery at the time of angiography.

### Statistical analyses

We examined EPA, DHA, EPA + DHA, and EPA/DHA ratios for their associations with MACE outcomes. The metabolites were examined based on quartiles, with the lowest level of 25% (the first quartile) as the reference category. The ratio of EPA to DHA was examined using a dichotomous threshold indicating more EPA than DHA (i.e., ratio >1) and less or equal amounts of EPA to DHA (i.e., ratio ≤1); a cut point of 1 for this ratio was chosen based on the nearest best fit by recursive partitioning (22).

The associations between the baseline EPA and DHA metabolite levels and 10-year MACE outcomes were examined using the Cox proportional hazard regression model with a censor for those with shorter follow-up time. Modeling was done in steps, with each metabolite examined first unadjusted, then adjusted for one another (e.g., EPA adjusted for DHA and vice-versa), and, finally, fully adjusted for other factors, e.g., age, sex, and comorbidities which are significantly associated with the metabolites. For significant comorbidities that highly correlated (*r* > 0.3), the variable deemed to be the most clinically significant based on the authors’ judgment was kept. As cholesterol and lipid values were missing in >10% of cases, we did not adjust for this in the modeling. However, we examined quartiles of triglycerides (the only lipid strongly associated with EPA and DHA) in a stratified subgroup analysis for the 10-year MACE risk. The additional subgroups examined in the stratified analysis for 10-year MACE were as follows: no or moderate CAD (stenosis <70%), severe CAD (i.e., at least one stenosis in a coronary artery or its major branch ≥70%), age (<60, 60–74, ≥75), sex, and prior heart failure. All statistical analyses were performed using SAS version 9.4. Significance was set at 0.01 for the comparisons of the primary endpoints, which were chosen as comparisons of the fourth quartile to the first quartile and per-quartile hazard ratios of fully adjusted EPA and DHA.

## Results

Of the 1,000 randomly selected samples, 994 were sent for metabolomic testing, of which 987 passed the quality filter thereafter (20, 21). Thus, the final cohort size for this study was 987. The average age was 61.5 ± 12 years, 57% were male, and 41% were obese. The subjects demonstrated a high burden of cardiovascular risk factors (Table 1). At angiography, 39% had no/mild CAD (<10% stenosis), 19% had moderate CAD (10%–69% stenosis), and 42% had severe CAD (≥70% stenosis of a coronary artery or major branch). A few differences were noted in baseline characteristics by baseline EPA and DHA levels (Supplementary Tables S1, S2, respectively). The significant baseline characteristics associated with EPA were hyperlipidemia, history of heart failure, chronic obstructive pulmonary disease (COPD), triglycerides, CAD severity, and percutaneous coronary intervention (PCI) (note: CAD severity and PCI were correlated, *r* = 0.51). The significant baseline characteristics associated with DHA were age, COPD, triglycerides, high-density lipoprotein cholesterol, and percutaneous coronary intervention (PCI).

**Table 1:** Patient and clinical characteristics of the study population (*n* = 987) at the time of angiography and sample ascertainment.

Age, mean ± SD	61.5 ± 12.15
Male, no. (%)	564 (57.1)
Obese (BMI ≥ 30), no. (%)	408 (41.3)
Smoking, no. (%)	
Never	734 (74.4)
Former	133 (13.5)
Current	120 (12.2)
Diabetic, no. (%)	320 (32.4)
Hx of hypertension, no. (%)	609 (61.7)
Hx of hyperlipidemia, no. (%)	536 (54.3)
Hx of heart failure, no. (%)	67 (6.8)
Hx of AF, no. (%)	108 (10.9)
Hx of COPD, no. (%)	113 (11.4)
Hx of stroke, no. (%)	20 (2.0)
Hx of depression, no. (%)	151 (15.3)
Family history of CVD, no. (%)	439 (44.5)
Prior statin use, no. (%)	306 (31.0)
Lipids	
Total cholesterol, mg/dl (*n* = 879)	181.5 ± 42.2
LDL-C, mg/dl (*n* = 828)	106.6 ± 34.2
HDL-C, mg/dl (*n* = 857)	42.4 ± 13.3
Triglycerides, mg/dl (*n* = 855)	163.9 ± 111.4
CAD at angiography, no. (%)	
No CAD	383 (38.8)
Mild/moderate CAD	191 (19.4)
Severe CAD	413 (41.8)
PCI performed, no. (%)	246 (24.9)

SD, standard deviation; no., number; BMI, body mass index; Hx, history; AF, atrial fibrillation; COPD, chronic obstructive pulmonary disease; CVD, cardiovascular disease; LDL-C, low-density lipoprotein cholesterol; HDL-C, high-density lipoprotein cholesterol; CAD, coronary artery disease; PCI, percutaneous coronary intervention.

### Ten-year MACE outcomes for EPA and DHA

**Figure 1:**
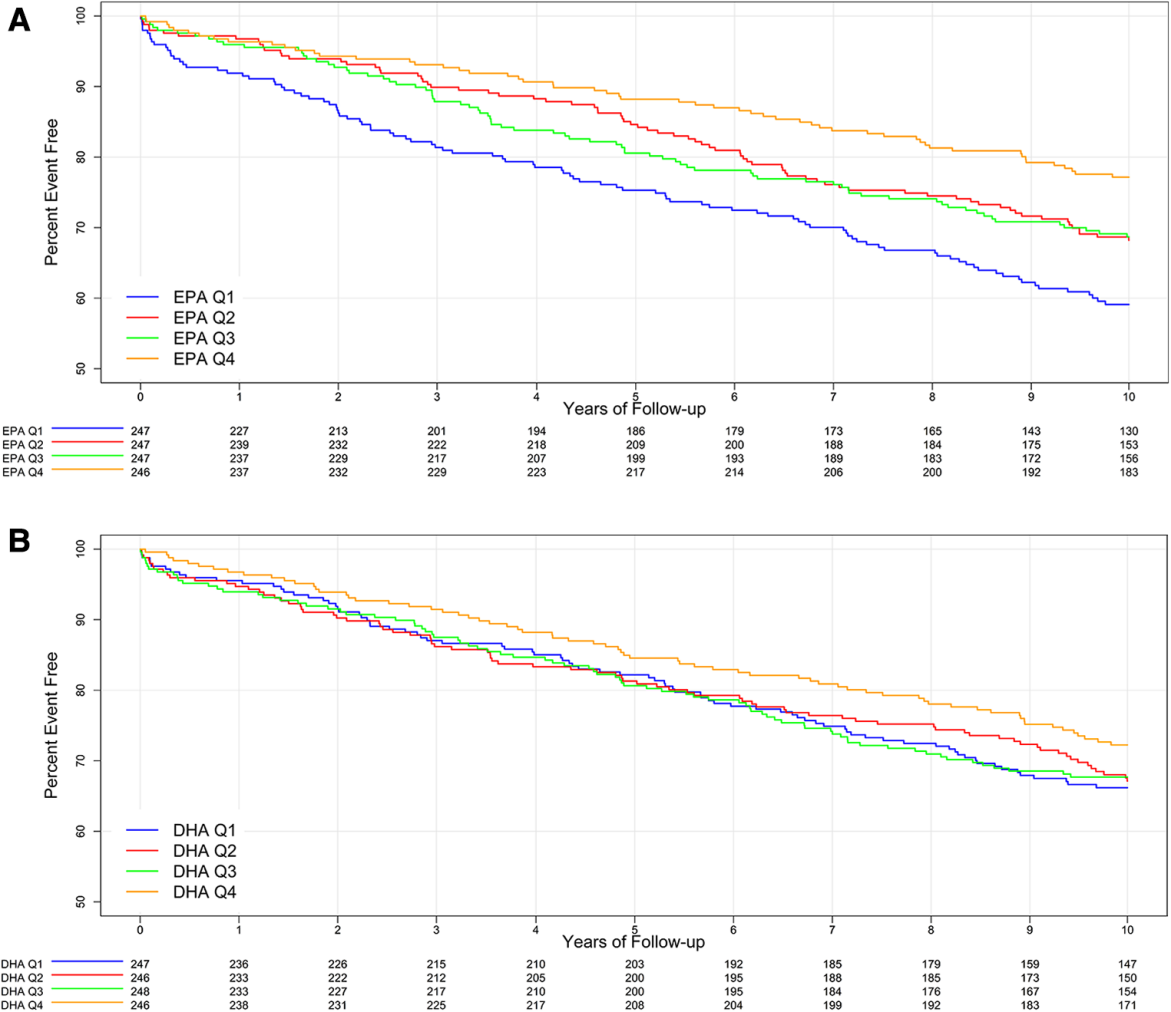
Kaplan–Meier curve for 10-year MACE outcomes for baseline EPA quartiles (**A**) and DHA quartiles (**B**).

The mean follow-up time was 12 ± 5 years. A total of 311 subjects (31.5%) had a MACE within 10 years, with an average time to the first MACE of 4.5 ± 3 years. The 10-year MACE rate was significantly associated with lower levels of EPA (unadjusted *p* = 0.0002, adjusted *p* = 0.0005) but not DHA (unadjusted *p* = 0.43, adjusted *p* = 0.52) (Table 2 and Figure 1). Subjects in the lowest (first) quartile of EPA had a 10-year MACE frequency of 40.5% compared to a 22.5% frequency for the highest (fourth) EPA quartile, with an absolute difference of 17.7%. The majority (75%) of these MACE were all-cause death (*n* = 234, 23.7% of subjects). The all-cause death rate was the only individual component of MACE that was significantly associated with EPA (unadjusted *p* < 0.0001, adjusted *p* = 0.0003).

**Table 2:** Ten-year MACE outcomes by baseline EPA and DHA quartiles. The mean time to the first MACE was 4.5 ± 3 years.

	EPA								** **	** **
	Q1		Q2		Q3		Q4		Unadjusted *p*-values	Adjusted *p*-values
10-year outcomes	*N* = 247		*N* = 247		*N* = 247		*N* = 246			
	Follow-up time 8.0 ± 3.2 years		Follow-up time 8.9 ± 2.3 years		Follow-up time 9.0 ± 2.3 years		Follow-up time 9.3 ± 2.1 years			
Follow-up time										
MACE	100	40.5%	78	31.6%	77	31.2%	56	22.8%	0.0002	0.0005
Death	87	35.2%	61	24.7%	53	21.5%	33	13.4%	<0.0001	0.0003
MI (>60 days)	4	1.6%	11	4.5%	7	2.8%	10	4.1%	0.61	0.74
Stroke	15	6.1%	13	5.3%	21	8.5%	13	5.3%	0.26	0.84
Heart failure Admission	11	4.5%	15	6.1%	13	5.3%	8	3.3%	0.15	0.26
	DHA									
	Q1		Q2		Q3		Q4			
	*N* = 247		*N* = 246		*N* = 248		*N* = 246		Unadjusted *p*-values	Adjusted *p*-values
	Follow-up time 8.5 ± 2.7 years		Follow-up time 8.7 ± 2.6 years		Follow-up time 8.8 ± 2.5 years		Follow-up time 9.1 ± 2.2 years			
MACE	83	33.6%	80	32.5%	80	32.3%	68	27.6%	0.43	0.52
Death	72	29.2%	63	25.6%	57	23.0%	42	17.1%	0.02	0.92
MI (>60 days)	6	2.4%	9	3.7%	8	3.2%	9	3.7%	0.90	0.57
Stroke	13	5.3%	12	4.9%	25	10.1%	12	4.9%	0.03	0.16
Heart failure Admission	10	4.1%	13	5.3%	11	4.4%	13	5.3%	0.51	0.99

EPA, eicosapentaenoic acid; DHA, docosahexaenoic acid; Q1, first quartile; Q2, second quartile; Q3, third quartile; Q4, forth quartile; MACE, major cardiovascular adverse events; MI, myocardial infarction. The Cox proportional hazard regression model was used for both unadjusted and adjusted *p*-values. Adjustments were made for age, gender, and significant comorbidities (EPA: hyperlipidemia, COPD, heart failure, and severe CAD; DHA: COPD and PCI performed).

**Figure 2:**
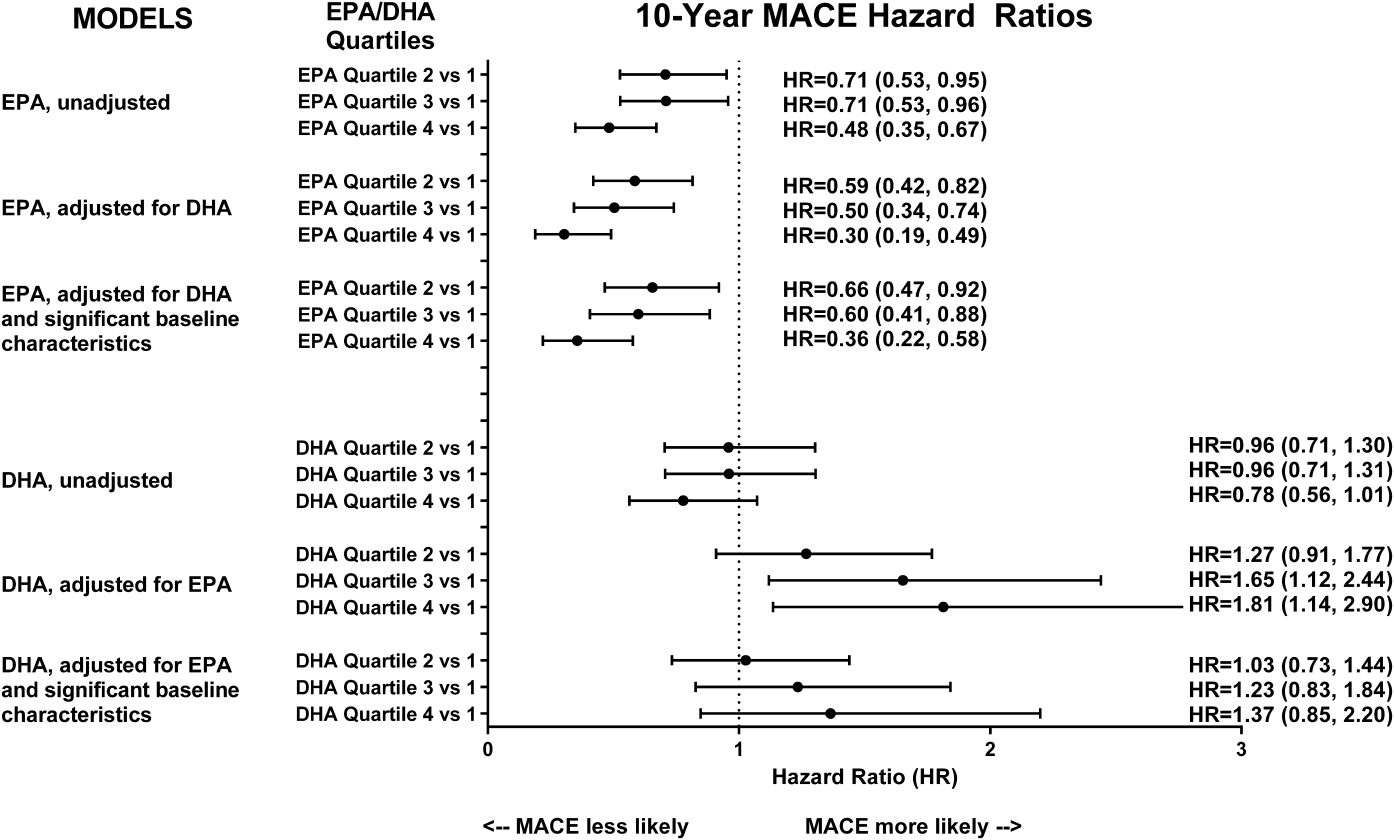
Hazard ratios (unadjusted and adjusted) for 10-year MACE among quartiles of baseline EPA and DHA.

All EPA analyses, unadjusted, adjusted for DHA, and adjusted for both DHA and baseline characteristics, found that higher EPA levels were statistically associated with a lower risk of MACE across higher (second–fourth) quartiles compared to the lowest (first) quartile (Figure 2). While the unadjusted hazard ratio (HR) of MACE for the highest (fourth) quartile of EPA was 0.48 (95% CI: 0.35, 0.67) compared to the lowest quartile, the adjustment for DHA improved the HR to 0.36 (95% CI: 0.22, 0.58), suggesting an adverse effect modification by DHA. Conversely, unadjusted DHA was non-significant. Furthermore, while adjustment for EPA resulted in the highest quartile of DHA, when compared to the lowest with a 1.81-fold higher risk of 10-year MACE (95% CI: 1.14, 2.90), the adjustment for additional baseline differences resulted in the DHA being not significantly associated with 10-year MACE. When analyzed by per-quartile risk change (Supplementary Table S3), the HR for EPA adjusted for DHA and baseline characteristics was 0.74 (95% CI: 0.63, 0.86), *p* < 0.0001, and the HR for DHA adjusted for EPA and baseline characteristics was 1.10 (95% CI: 0.94, 1.28), *p* = 0.22.

For EPA, most subgroup analyses had similar HRs as the overall adjusted HR (Supplementary Figure S1). When stratified by CAD severity, EPA Q4 and Q1 among both severe CAD patients [adjusted HR = 0.35 (95% CI: 0.18, 0.68)] and those with no, mild, or moderate CAD [adjusted HR = 0.42 (95%CI: 0.20, 0.85)] were significantly associated with 10-year MACE. Prior heart failure, age ≥75, and TG ≥200 showed no significant association with 10-year MACE for all EPA quartile comparisons.

For DHA, most subgroup analyses showed non-significant associations with 10-year MACE (Supplementary Figure S2). However, for patients <60, HRs for DHA Q3 and Q4 compared to those for DHA Q1 were significantly associated with an increased risk of 10-year MACE [Q3 vs. Q1 adjusted HR = 2.89 (95%CI: 1.34, 6.21) and Q4 vs. Q1 adjusted HR = 3.95 (95%CI: 1.56, 10.00)].

### EPA + DHA

The significant baseline characteristics associated with EPA + DHA composite were age, hyperlipidemia, COPD, high-density lipoprotein cholesterol, triglycerides, CAD severity, and PCI. After adjustment, the EPA + DHA composite was significantly associated with a decreased 10-year MACE risk, *p* < 0.0001 (Supplementary Table S5). The 10-year MACE adjusted HRs for EPA + DHA Q3 vs. Q1 and Q3 vs. Q1 were 0.67 (95% CI: 0.49, 0.91) and 0.52 (95% CI: 0.37, 0.72), respectively. EPA + DHA was significantly associated with death (*p* < 0.0001) but none of the other MACE outcome components (Supplementary Table S5). The subgroup analyses provided similar HRs (Supplementary Figure S3).

### EPA/DHA ratio

**Figure 3:**
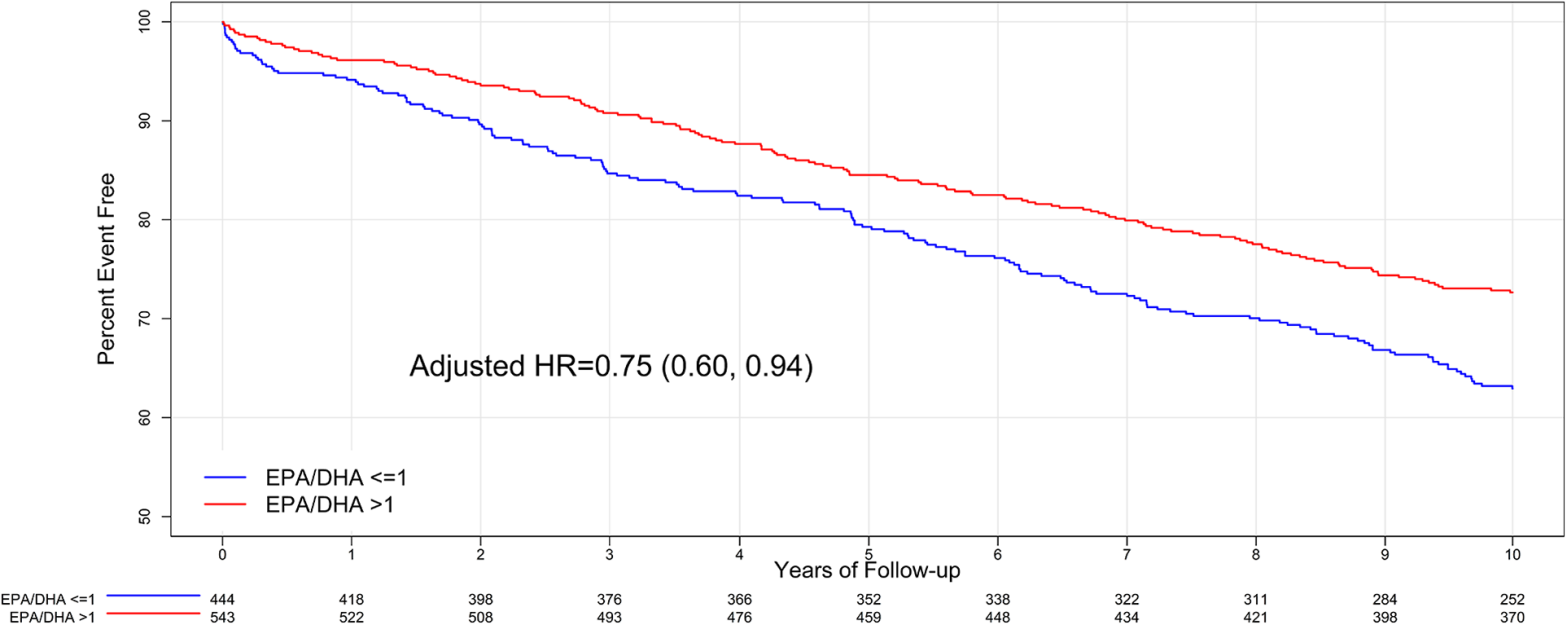
Kaplan–Meier curve for 10-year MACE outcomes for baseline EPA/DHA ratio.

A total of 444 (45%) subjects had an EPA/DHA ratio of ≤1, and 543 subjects (55%) had an EPA/DHA ratio of >1. The baseline characteristics of these two EPA/DHA ratio groups were compared (Supplementary Table S6), and the significant baseline characteristics associated with the EPA/DHA ratio were hyperlipidemia, COPD, family history of CAD, prior statin use, and PCI. EPA/DHA >1 was associated with a lower risk of 10-year MACE compared to EPA/DHA ≤1 (27% vs. 37%, respectively, and unadjusted *p*-value = 0.001). However, after adjustment for baseline differences, this decrease was not significant given the multiple testing corrections (adjusted *p*-value = 0.013) (Supplementary Table S7). The adjusted HR for 10-year MACE was 0.75 (95% CI: 0.60, 0.94) for an EPA/DHA ratio >1 compared to an EPA/DHA ≤1 (Figure 3). Subgroup analyses for EPA/DHA were similar to the overall results (Supplementary Table S8).

## Discussion

We found that higher plasma levels of EPA and EPA + DHA combined were associated with a protective effect for incident MACE. However, unadjusted DHA itself was not associated with incident MACE or a protective effect. Furthermore, DHA adjusted for EPA resulted in almost a 2-fold increased risk of MACE for the highest compared to the lowest quartile of DHA. Further, DHA appeared to reduce the effect of EPA, such that the adjustment for DHA resulted in a larger estimated protective effect of higher EPA plasma levels. The relationship between EPA and DHA was further illustrated by the analyses of the EPA/DHA ratio, which showed a lower risk of 10-year MACE with EPA/DHA ratios >1 (i.e., higher EPA and DHA plasma levels). These findings may have been unanticipated given prior smaller, shorter-duration studies, which suggested the opposite, where DHA was associated with a greater impact on biomarkers (e.g., inflammatory ones), lipoproteins, heart rate, and blood pressure than EPA (23–32). However, they are consistent with more recent, large, randomized intervention trials.

Our finding of a protective effect of EPA and EPA + DHA circulating plasma levels on MACE was consistent with those of Harris et al., even though we did not find an association with DHA circulating plasma levels alone (9). Importantly, the protective effect of EPA remained after adjusting for DHA, which in fact demonstrated a more pronounced effect when associated with lower DHA levels and decreasing with higher levels of DHA. The EPA + DHA analysis benefited from the statistically significant impact of EPA Q4 on MACE, whether EPA is unadjusted or adjusted. Unadjusted DHA in our analysis was not statistically significant with regard to an impact on MACE, although a numerical trend was noted toward lower events in those in Q4. However, when adjusted for associated EPA levels, with or without other adjustments for other comorbidities, this trend reversed toward harm.

Altogether, these observations are consistent with a proposed anti-inflammatory/membrane-stabilizing effect of EPA, which, in part, is neutralized by increasing levels of DHA. In contrast, DHA appears to be devoid of significant protective effects and may detrimentally impact or blunt the protection conferred by elevated EPA levels, in particular when the EPA/DHA ratio is ≤1.

Our findings of a protective effect of EPA circulating levels and the adverse interplay with DHA both complemented and helped to explain the mixed results of the published clinical trials. Two randomized trials, the Japanese JELIS trial and the international REDUCE-IT trial, found cardioprotection from EPA alone. The JELIS trial of 18,645 hypercholesterolemic patients at primary or secondary risk for cardiovascular events found that 1,800 mg of EPA daily with a statin compared to a statin alone reduced the primary MACE endpoint by 19% (*p* = 0.011) (33). The baseline EPA level was 93 mcg/ml in the standard group and 97 mcg/ml in the treatment group, which was increased to 170 mcg/ml in those who received 1,800 mg of EPA (34, 35). Similarly, the REDUCE-IT trial of statin-treated patients with triglycerides between 135 mg/dl and 500 mg/dl and a history of ASCVD or diabetes (*n* = 8,179) found that icosapent ethyl (4 g/day), a high potency source of EPA, reduced the first MACE by 25% and the total MACE by 30% (*p* < 0.0001) compared to a placebo of mineral oil (7, 8). The median observed EPA level was 26.1 mcg/ml in both groups, which increased to a median of 150 mcg/ml at the last visit in those receiving 4 g/day of EPA (11). The REDUCE-IT findings led to the FDA approval of icosapent ethyl as an adjunctive therapy in cardiovascular risk reduction in those with triglyceride levels ≥150 mg/dl and established cardiovascular disease or diabetes with two or more risk factors and its incorporation into several practice guidelines (36–39). Our study found that having EPA levels in the highest quartile compared to the lowest showed an absolute reduction in 10-year MACE of 17.7% and resulted in an adjusted HR of 0.30, indicating a 70% relative risk reduction in 10-year MACE. Our samples were analyzed *via* liquid chromatography-mass spectrometry and reported in ASU. There were only 100 plasma samples sent to Boston Heart Diagnostics, Framingham, MA, USA, for traditional OM3 fatty acid levels. The means for EPA and DHA serum concentrations were 20.42 ± 10.17 mcg/ml and 52.14 ± 17.58 mcg/ml, respectively, and were somewhat lower than the median baseline EPA levels in both STRENGTH and REDUCE-IT (21.0 and 26.1, respectively) as well as reported in a representative US cohort with baseline characteristics similar to JELIS (median <50 mcg/ml) (11, 14, 35). Our data were analyzed in quartiles using ASU, and this does not allow for definitive comparisons to either the baseline or achieved levels of plasma EPA levels seen in CVOTs.

In contrast, four other supplement trials using mixtures of OM3s, including both EPA and DHA, failed to find a reduction in MACE. Both VITAL, the largest primary prevention trial (*n* = 25,871), and ASCEND (*n* = 15,480 patients with diabetes but no evidence of cardiovascular disease) used mixed (EPA and DHA) marine OM3 PUFAs (1 g/day) compared to placebo (12, 13). Neither study found a significant lowering in incident MACE. The STRENGTH trial (4 g/day or placebo; *n* = 13,078 patients with triglycerides ≥180 and <500 mg/dl) was terminated early due to a low likelihood of demonstrating a risk reduction. Finally, the Norwegian OMEMI trial (*n* = 1,027 patients aged 70–82 years who experienced a recent acute MI) also found no reduction in MACE from combined EPA and DHA supplementation (15).

As far as we know, our study is one of the first to demonstrate that DHA may blunt the cardioprotective effect of EPA. Thus, as these latter trials used compounds that contained both EPA and DHA, our findings can help account for some of the difference in their results from that of the REDUCE-IT and JELIS EPA-only trials (11, 33). Other potential contributors offered for these differences have included differing populations; study designs and endpoints; choice of placebo (corn oil vs. mineral oil); and the play of chance, which should be addressed in additional investigations.

In REDUCE-IT, on-treatment EPA levels correlated strongly with total and individual MACE endpoints and did so independently of baseline and achieved triglyceride levels (40). The REDUCE-IT investigators argued that their trial data provide a mechanistic explanation for risk reductions beyond those predicted by the reductions in triglycerides that were observed with EPA supplementation. This was further supported by the EVAPORATE trial, which enrolled a similar cohort to REDUCE-IT and examined the progression of coronary atherosclerosis by coronary computed tomographic angiography in 80 patients with elevated triglycerides on statin therapy and randomized to icosapent ethyl (EPA 4 g/day) or placebo (41). In an intention-to-treat analysis, the primary endpoint was achieved, with reduction in low-attenuation plaque being statistically significant in the treatment group vs. the placebo group (−0.3 ± 1.5 vs. 0.9 ± 1.7 mm^3^; *p* = 0.006). Changes in total non-calcified plaque, total plaque, and fibrous plaque were also significantly reduced compared to placebo, providing further mechanistic support for a direct effect on atherosclerotic plaque, which could underlie the risk reduction seen in the larger REDUCE-IT trial with EPA-only therapy. A meta-analysis by Fan et al. (42), comparing OM3 PUFA added to statin vs. statin alone on coronary plaques, included EVAPORATE and seven other studies, three of which had EPA/DHA combination arms. Fan et al. concluded that “the combination of OM3 and statins may be superior to statin treatment alone.” Of the three studies with an EPA/DHA combination arm, only the study by Alfaddagh et al. (43) concluded that there was an additional benefit in using EPA/DHA combination therapy added to statin in preventing fibrous coronary plaque (44, 45). However, the primary endpoint of reduction in non-calcified plaque volume was not significant on an intention-to-treat analysis (*p* = 0.14) and only achieved significance on a per-protocol analysis, *p*-0.07 (44, 45).

Recent reviews have summarized the contrasting underlying molecular mechanisms of EPA and DHA and provide further support for our findings of the protective effect of EPA and blunting of that protection by DHA (46, 47). EPA acts to preserve cellular membrane structure and distribution of cholesterol, inhibits lipid oxidation and cholesterol crystalline domain formation, and impacts signal transduction pathways related to inflammation and vasodilation (48–50). In contrast, DHA increases membrane fluidity, promotes lipid domain changes, reduces antioxidant activity in association with its lipid-disordering effects, and is concentrated primarily in brain and retinal membranes. Beyond the differences between EPA and DHA in the membrane lipid layer (50), EPA and DHA molecules directly compete for incorporation into the cell membrane by differentially displacing omega-6 PUFAs, affecting downstream metabolites involved in the initiation and resolution of inflammation (46). There are also differences between the antioxidant effects of EPA and DHA, which could have clinical ramifications. EPA and DHA both have been shown *in vitro* to reduce the oxidations of apoB-containing particles, including sdLDL, VLDL, LDL, and also HDL; however, EPA's antioxidant effects were more prolonged (51, 52). Finally, DHA has been shown to lower the conversion rate of EPA to its longer-chain form of docosapentaenoic acid (46).

As noted, EPA impacts inflammation pathways, directly competing with arachidonic acid (AA), a fatty acid that is a precursor to proinflammatory agents: prostaglandins, thromboxane A2, and leukotrienes (53). EPA/AA ratios are associated with cardiovascular events, higher ratios (increased EPA levels) whether baseline or achieved, with lower events (54, 55). EPA/AA ratios (<0.4) were utilized for randomization criteria in the open-label CVOT follow-up to the JELIS trial, RESPECT-EPA (56). We previously reported preliminary data as an abstract showing a statistically significant association between EPA/AA ratios and MACE for the EPA only Q4 group for both univariate and multivariate analyses, HR = 0.560 (95% CI: 0.419, 0.748) and HR = 0.565 (95% CI: 0.422, 0.758), respectively, with similar *p*-values of 0.0001 (57). While a statistically significant association was seen, this was only in the EPA Q4 group and does not add to the current analysis of EPA and DHA interaction.

Our results, together with observations from REDUCE-IT and EVAPORATE, provide a mechanistic understanding of both earlier and contemporary divergent trial results and suggest implications for clinical practice. Our data add importantly to an increasing consensus that achieving therapeutic EPA (e.g., achieved EPA level, 169 mcg/ml in JELIS and 150 mcg/ml in REDUCE-IT) but not DHA levels is necessary for cardiovascular protection. For EPA-deficient subjects (e.g., baseline EPA level, 26.1 mcg/ml in REDUCE-IT), which may represent a large percentage of the US population (11), prescriptive medication rather than off-the-shelf supplements appears to be necessary. Indeed, our observations in a population not specifically being treated with OM3 demonstrate that achieving higher “prescriptive” levels of DHA may interfere with the MACE-reducing benefit of EPA. Thus, whether DHA-containing mixed OM3 products, prescription or off-the shelf, detract from OM3 benefits is a prime topic for further study.

Our study has several limitations that are common with observational studies. First, the associations with outcomes may be confounded by differences in other co-linear fatty acid metabolites. Similarly, unaccounted baseline characteristics might impact these results; however, it is reassuring that adjustments for known baseline differences had little impact on the findings. Furthermore, lipid values were missing in >10% of the subjects, so we did not include this in the adjustments. However, to ensure this, we performed a sensitivity analysis of the adjusted results adding triglycerides as a confounder. These results were similar to the adjusted results without triglycerides, and the over-conclusions were unchanged. Another limitation is that information on subjects’ diets, supplements, and prescription medications was not available. However, the EPA levels should, mechanistically, more directly affect cardiovascular risk associations regardless of how they were attained, and the meta-analyses by Harris et al. (9) and Chowdhury et al. (58) both found inverse relationships between cardiovascular outcomes and EPA, DHA, and EPA + DHA circulating levels, unadjusted for each other. We analyzed the MACE outcomes in relation to an index plasma level of EPA and DHA; however, we do not have data on whether these changed over time and whether such a change influenced our MACE outcomes. We did not have the cause of death, so we, therefore, examined all-cause mortality. Thus, we were not able to specifically differentiate the impact on cardiovascular-related deaths. However, the use of all-cause mortality provided a hard and relevant overall endpoint, and prior studies of patients with heart disease found that at least 40%–60% of the deaths were related to cardiovascular causes (59–61). Finally, these findings were limited to a single center and a population of primarily white subjects of European ancestry and may not apply to other ethnic/racial groups. Despite these limitations, this was a large, long-term prospectively enrolled registry study with coronary angiography, to define baseline coronary disease extent, and high-pressure liquid chromatography, to precisely define relative levels of EPA and DHA.

## Conclusion

The INSPIRE biobank registry provided a unique opportunity to examine the relationship between spontaneously achieved levels of OM3 metabolites to incident long-term MACE among a mix of high-risk primary and secondary prevention populations referred for angiography. These results supported the observed protective effect of circulating and achieved higher levels of EPA in EPA-only RCT CVOT, but not necessarily DHA, on incident MACE. More importantly, these results suggested that higher DHA levels and a resulting lower EPA/DHA ratio may blunt the cardioprotective effectiveness of EPA. These results in conjunction with lower achieved EPA serum levels may help explain the neutral findings in some of the recent CV outcome trials, including the STRENGTH and OMEMI trials.

## Data Availability

The datasets presented in this article are not readily available because the data underlying this article cannot be shared publicly due to US privacy laws. Data are available upon reasonable request to the corresponding author. Requests to access the datasets should be directed to viet.le@imail.org.
