# Supplementary material for: Higher docosahexaenoic acid levels lower the protective impact of eicosapentaenoic acid on long-term major cardiovascular events
Source: Front Cardiovasc Med. 2023 Aug 23;10:1229130. doi: 10.3389/fcvm.2023.1229130 (PMC10482040; doi:10.3389/fcvm.2023.1229130)
Supplement: Supplementary file 1 [file Table1.docx]

**Supplementary Tables and Figures**

**Supplementary Table 1:** Baseline characteristics by baseline EPA quartiles

|  | **EPA** | | | | | | | |  |
| --- | --- | --- | --- | --- | --- | --- | --- | --- | --- |
|  | **Q1** | | **Q2** | | **Q3** | | **Q4** | |  |
|  | N=247 | | N=247 | | N=247 | | N=246 | | **Pvalue** |
| **Age, mean ± std** | 60.7 ± 11.6 | | 60.8 ± 13.2 | | 62.3 ± 12.0 | | 62.3 ± 11.6 | | 0.18 |
| **Male, No. (%)** | 141 | (57.1%) | 145 | (58.7%) | 125 | (50.6%) | 153 | (62.2%) | 0.07 |
| **Obese (BMI≥30), No. (%)** | 99 | (40.1%) | 101 | (40.9%) | 115 | (46.6%) | 93 | (37.8%) | 0.24 |
| **Smoking History** |  |  |  |  |  |  |  |  | 0.95 |
| Never | 182 | (73.7%) | 180 | (72.9%) | 188 | (76.1%) | 184 | (74.8%) |  |
| Former | 32 | (13.0%) | 38 | (15.4%) | 31 | (12.6%) | 32 | (13.0%) |  |
| Current | 33 | (13.4%) | 29 | (11.7%) | 28 | (11.3%) | 30 | (12.2%) |  |
| **Diabetic, No. (%)** | 85 | (34.4%) | 77 | (31.2%) | 83 | (33.6%) | 75 | (30.5%) | 0.75 |
| **Hx of Hypertension, No. (%)** | 157 | (63.6%) | 146 | (59.1%) | 167 | (67.6%) | 139 | (56.5%) | 0.06 |
| **Hx of Hyperlipidemia, No. (%)** | 106 | (42.9%) | 132 | (53.4%) | 148 | (59.9%) | 150 | (61.0%) | 0.0001 |
| **Hx of Heart Failure, No. (%)** | 26 | (10.5%) | 9 | (3.6%) | 15 | (6.1%) | 17 | (6.9%) | 0.02 |
| **Hx of AF, No. (%)** | 27 | (10.9%) | 31 | (12.6%) | 26 | (10.5%) | 24 | (9.8%) | 0.79 |
| **Hx of COPD, No. (%)** | 36 | (14.6%) | 37 | (15.0%) | 23 | (9.3%) | 17 | (6.9%) | 0.01 |
| **Hx. of Stroke, No. (%)** | 3 | (1.2%) | 7 | (2.8%) | 6 | (2.4%) | 4 | (1.6%) | 0.57 |
| **Hx of Depression, No. (%)** | 30 | (12.1%) | 40 | (16.2%) | 48 | (19.4%) | 33 | (13.4%) | 0.11 |
| **Family History of CAD, No. (%)** | 107 | (43.3%) | 99 | (40.1%) | 111 | (44.9%) | 122 | (49.6%) | 0.20 |
| **Prior Statin Use, No. (%)** | 69 | (27.9%) | 80 | (32.4%) | 84 | (34.0%) | 73 | (29.7%) | 0.47 |
| **Lipids** |  |  |  |  |  |  |  |  |  |
| Total Cholesterol, mg/dL (n=879) | 174.5 ± 38.4 | | 181.1 ± 40.8 | | 183.5 ± 39.2 | | 187.1 ± 49.3 | | 0.08 |
| LDL-C, mg/dL (n=828) | 104.1 ± 31.9 | | 108.8 ± 33.9 | | 106.4 ± 33.6 | | 107.0 ± 37.4 | | 0.70 |
| HDL-C, mg/dL (n=857) | 40.9 ± 12.9 | | 41.8 ± 11.9 | | 43.8 ± 14.9 | | 42.9 ± 13.4 | | 0.36 |
| Triglycerides, mg/dL (n=855) | 145.3 ± 91.8 | | 157.4 ± 103.1 | | 165.5 ± 100.4 | | 189.1 ± 142.9 | | 0.002 |
| **CAD** |  |  |  |  |  |  |  |  | <0.0001 |
| No CAD | 98 | (39.7%) | 113 | (45.7%) | 108 | (43.7%) | 64 | (26.0%) |  |
| Mild/Moderate CAD | 53 | (21.5%) | 43 | (17.4%) | 50 | (20.2%) | 45 | (18.3%) |  |
| Severe CAD | 96 | (38.9%) | 91 | (36.8%) | 89 | (36.0%) | 137 | (55.7%) |  |
| **PCI Performed** | 44 | (17.8%) | 47 | (19.0%) | 62 | (25.1%) | 93 | (37.8%) | <0.0001 |

Note: EPA= eicosapentaenoic acid, Q1=first quartile, Q2=second quartile, Q3=third quartile, Q4=forth quartile, std=standard deviation, No.=number, BMI=body mass index, Hx=history, AF=atrial fibrillation, COPD= chronic obstructive pulmonary disease, CVD=cardiovascular disease, LDL-C= low-density lipoprotein cholesterol, HDL-C= high-density lipoprotein cholesterol, CAD=coronary artery disease, PCI= percutaneous coronary intervention. P-values from Kruskal–Wallis test (for continuous variables), Pearson chi-square tests (for categorical variables) and Fisher exact test (for categorical variables with small cell sizes).

**Supplementary Table 2:** Baseline characteristics by baseline DHA quartiles

|  | **DHA** | | | | | | | |  |
| --- | --- | --- | --- | --- | --- | --- | --- | --- | --- |
|  | **Q1** | | **Q2** | | **Q3** | | **Q4** | |  |
|  | N=247 | | N=246 | | N=248 | | N=246 | | **Pvalue** |
| **Age, mean ± std** | 57.6 ± 12.5 | | 61.5 ± 12.0 | | 63.1 ± 11.7 | | 63.0 ± 12.0 | | <0.0001 |
| **Male, No. (%)** | 152 | (61.5%) | 136 | (55.3%) | 144 | (58.1%) | 132 | (53.7%) | 0.31 |
| **Obese (BMI>=30), No. (%)** | 106 | (42.9%) | 111 | (45.1%) | 102 | (41.1%) | 89 | (36.2%) | 0.22 |
| **Smoking History** |  |  |  |  |  |  |  |  | 0.72 |
| Never | 175 | (70.8%) | 180 | (73.2%) | 188 | (75.8%) | 191 | (77.6%) |  |
| Former | 37 | (15.0%) | 36 | (14.6%) | 32 | (12.9%) | 28 | (11.4%) |  |
| Current | 35 | (14.2%) | 30 | (12.2%) | 28 | (11.3%) | 27 | (11.0%) |  |
| **Diabetic, No. (%)** | 82 | (33.2%) | 80 | (32.5%) | 76 | (30.7%) | 82 | (33.3%) | 0.91 |
| **Hx of Hypertension, No. (%)** | 152 | (61.5%) | 152 | (61.8%) | 152 | (61.3%) | 153 | (62.2%) | 1.00 |
| **Hx of Hyperlipidemia, No. (%)** | 119 | (48.2%) | 134 | (54.5%) | 142 | (57.3%) | 141 | (57.3%) | 0.14 |
| **Hx of Heart Failure, No. (%)** | 19 | (7.7%) | 12 | (4.9%) | 17 | (6.9%) | 19 | (7.7%) | 0.56 |
| **Hx of AF, No. (%)** | 20 | (8.1%) | 25 | (10.2%) | 38 | (15.3%) | 25 | (10.2%) | 0.06 |
| **Hx of COPD, No. (%)** | 32 | (13.0%) | 36 | (14.6%) | 28 | (11.3%) | 17 | (6.9%) | 0.046 |
| **Hx. of Stroke, No. (%)** | 2 | (0.8%) | 6 | (2.4%) | 7 | (2.8%) | 5 | (2.0%) | 0.42 |
| **Hx of Depression, No. (%)** | 34 | (13.8%) | 47 | (19.1%) | 28 | (11.3%) | 42 | (17.1%) | 0.08 |
| **Family History of CAD, No. (%)** | 103 | (41.7%) | 114 | (46.3%) | 100 | (40.3%) | 122 | (49.6%) | 0.14 |
| **Prior Statin Use, No. (%)** | 79 | (32.0%) | 79 | (32.1%) | 74 | (29.8%) | 74 | (30.1%) | 0.92 |
| **Lipids** |  |  |  |  |  |  |  |  |  |
| Total Cholesterol, mg/dL (n=879) | 176.3 ± 39.9 | | 183.6 ± 36.9 | | 179.3 ± 42.9 | | 187.1 ± 48.2 | | 0.08 |
| LDL-C, mg/dL (n=828) | 105.0 ± 32.7 | | 110.7 ± 32.0 | | 104.1 ± 34.8 | | 106.2 ± 37.1 | | 0.09 |
| HDL-C, mg/dL (n=857) | 39.9 ± 12.1 | | 42.4 ± 13.5 | | 44.2 ± 14.5 | | 43.1 ± 12.9 | | 0.03 |
| Triglycerides, mg/dL (n=855) | 151.5 ± 90.1 | | 156.8 ± 104.7 | | 155.2 ± 100.1 | | 193.9 ± 141.4 | | 0.0005 |
| **CAD** |  |  |  |  |  |  |  |  | 0.22 |
| No CAD | 104 | (42.1%) | 102 | (41.5%) | 98 | (39.5%) | 79 | (32.1%) |  |
| Mild/Moderate CAD | 50 | (20.2%) | 48 | (19.5%) | 44 | (17.7%) | 49 | (19.9%) |  |
| Severe CAD | 93 | (37.7%) | 96 | (39.0%) | 106 | (42.7%) | 118 | (48.0%) |  |
| **PCI Performed** | 45 | (18.2%) | 52 | (21.1%) | 70 | (28.2%) | 79 | (32.1%) | 0.001 |

Note: DHA = docosahexaenoic acid, Q1=first quartile, Q2=second quartile, Q3=third quartile, Q4=forth quartile, std=standard deviation, No.=number, BMI=body mass index, Hx=history, AF=atrial fibrillation, COPD= chronic obstructive pulmonary disease, CVD=cardiovascular disease, LDL-C= low-density lipoprotein cholesterol, HDL-C= high-density lipoprotein cholesterol, CAD=coronary artery disease, PCI= percutaneous coronary intervention. P-values from Kruskal–Wallis test (for continuous variables), Pearson chi-square tests (for categorical variables) and Fisher exact test (for categorical variables with small cell sizes).

**Supplementary Table 3:** Hazard Ratios (unadjusted and adjusted) for 10-Year MACE per quartile higher* of baseline EPA and DHA

|  | **HR for 10-Year MACE, per quartile higher** | **95% Confidence Interval** | **p-value** |
| --- | --- | --- | --- |
| **EPA, unadjusted** | 0.81 | (0.73, 0.89) | <0.0001 |
| **EPA, adjusted for DHA** | 0.69 | (0.59, 0.80) | <0.0001 |
| **EPA, adjusted for DHA and baseline characteristics** | 0.74 | (0.63, 0.86) | <0.0001 |
| **DHA, unadjusted** | 0.93 | (0.84, 1.03) | 0.15 |
| **DHA, adjusted for EPA** | 1.23 | (1.06, 1.43) | 0.008 |
| **DHA, adjusted for EPA and baseline characteristics** | 1.10 | (0.94, 1.28) | 0.22 |

Note: EPA= eicosapentaenoic acid, DHA = docosahexaenoic acid, Q1=first quartile, Q2=second quartile, Q3=third quartile, Q4=forth quartile, MACE=major cardiovascular adverse events. Cox proportional hazard regression used for both unadjusted and adjusted p-values. Adjustment made for age, gender and significant comorbidities (EPA: hyperlipidemia, COPD, heart failure, and severe CAD; DHA: COPD and PCI performed).

* quartiles were treated as continuous variable in Cox proportional hazard regression.

**Supplementary Figure 1:** Subgroup analyses; adjusted HR for 10-MACE by baseline EPA quartiles.

Note: EPA= eicosapentaenoic acid, DHA = docosahexaenoic acid, MACE=major cardiovascular adverse events, CAD=coronary artery disease, HF=heart failure, TG=triglyceride, HR=hazard ratio. Cox proportional hazard regression used with adjustment made for DHA, age, gender and significant comorbidities (hyperlipidemia, COPD, heart failure, and severe CAD); variables removed from adjustment if used for subgroup stratification.

**Supplementary Figure 2:** Subgroup analyses; adjusted HR for 10-MACE by baseline DHA quartiles.

Note: EPA= eicosapentaenoic acid, DHA = docosahexaenoic acid, MACE=major cardiovascular adverse events, CAD=coronary artery disease, HF=heart failure, HR=hazard ratio. Cox proportional hazard regression used with adjustment made for EPA, age, gender and significant comorbidities (COPD and PCI performed); variables removed from adjustment if used for subgroup stratification.

**Supplementary Table 4:** Baseline characteristics by baseline EPA+DHA quartiles

|  | **EPA + DHA** | | | | | | | |  |
| --- | --- | --- | --- | --- | --- | --- | --- | --- | --- |
|  | **Q1** | | **Q2** | | **Q3** | | **Q4** | |  |
|  | N=247 | | N=247 | | N=246 | | N=247 | | **Pvalue** |
| **Age, mean ± std** | 59.7 ± 12.4 | | 61.7 ± 12.2 | | 62.3 ± 12.1 | | 62.5 ± 11.9 | | 0.04 |
| **Male, No. (%)** | 146 | (59.1%) | 143 | (57.9%) | 127 | (51.6%) | 148 | (59.5%) | 0.23 |
| **Obese (BMI≥30), No. (%)** | 101 | (40.9%) | 110 | (44.5%) | 107 | (43.5%) | 90 | (36.4%) | 0.26 |
| **Smoking History** |  |  |  |  |  |  |  |  | 0.38 |
| Never | 174 | (70.4%) | 191 | (77.3%) | 181 | (73.6%) | 188 | (76.1%) |  |
| Former | 37 | (15.0%) | 35 | (14.2%) | 31 | (12.6%) | 30 | (12.2%) |  |
| Current | 36 | (14.6%) | 21 | (8.5%) | 34 | (13.8%) | 29 | (11.7%) |  |
| **Diabetic, No. (%)** | 81 | (32.8%) | 85 | (34.4%) | 74 | (30.1%) | 80 | (32.4%) | 0.78 |
| **Hx of Hypertension, No. (%)** | 154 | (62.4%) | 147 | (59.5%) | 164 | (66.7%) | 144 | (58.3%) | 0.23 |
| **Hx of Hyperlipidemia, No. (%)** | 113 | (45.8%) | 126 | (51.0%) | 154 | (62.6%) | 143 | (57.9%) | 0.0009 |
| **Hx of Heart Failure, No. (%)** | 21 | (8.5%) | 16 | (6.5%) | 11 | (4.5%) | 19 | (7.7%) | 0.31 |
| **Hx of AF, No. (%)** | 22 | (8.9%) | 32 | (13.0%) | 31 | (12.6%) | 23 | (9.3%) | 0.33 |
| **Hx of COPD, No. (%)** | 38 | (15.4%) | 32 | (13.0%) | 29 | (11.8%) | 14 | (5.7%) | 0.006 |
| **Hx. of Stroke, No. (%)** | 2 | (0.8%) | 7 | (2.8%) | 5 | (2.0%) | 6 | (2.4%) | 0.41 |
| **Hx of Depression, No. (%)** | 35 | (14.2%) | 38 | (15.4%) | 46 | (18.7%) | 32 | (13.0%) | 0.32 |
| **Family History of CAD, No. (%)** | 105 | (42.5%) | 98 | (39.7%) | 120 | (48.8%) | 116 | (47.0%) | 0.16 |
| **Prior Statin Use, No. (%)** | 72 | (29.2%) | 77 | (31.2%) | 84 | (34.2%) | 73 | (29.6%) | 0.62 |
| **Lipids** |  |  |  |  |  |  |  |  |  |
| Total Cholesterol, mg/dL (n=879) | 175.1 ± 37.8 | | 182.9 ± 42.8 | | 180.1 ± 38.0 | | 188.3 ± 48.7 | | 0.06 |
| LDL-C, mg/dL (n=828) | 104.2 ± 30.8 | | 110.8 ± 36.0 | | 103.2 ± 32.5 | | 108.2 ± 36.9 | | 0.16 |
| HDL-C, mg/dL (n=857) | 40.8 ± 12.7 | | 41.5 ± 12.0 | | 44.9 ± 15.2 | | 42.3 ± 12.9 | | 0.04 |
| Triglycerides, mg/dL (n=855) | 148.7 ± 97.5 | | 156.8 ± 97.3 | | 159.6 ± 96.6 | | 192.9 ± 145.9 | | 0.001 |
| **CAD** |  |  |  |  |  |  |  |  | 0.016 |
| No CAD | 106 | (42.9%) | 109 | (44.1%) | 109 | (44.3%) | 81 | (32.8%) |  |
| Mild/Moderate CAD | 45 | (18.2%) | 47 | (19.0%) | 40 | (16.3%) | 37 | (15.0%) |  |
| Severe CAD | 96 | (38.9%) | 91 | (36.8%) | 97 | (39.4%) | 129 | (52.2%) |  |
| **PCI Performed** | 46 | (18.6%) | 45 | (18.2%) | 68 | (27.6%) | 87 | (35.2%) | <0.0001 |

Note: EPA= eicosapentaenoic acid, DHA = docosahexaenoic acid, Q1=first quartile, Q2=second quartile, Q3=third quartile, Q4=forth quartile, std=standard deviation, No.=number, BMI=body mass index, Hx=history, AF=atrial fibrillation, COPD= chronic obstructive pulmonary disease, CVD=cardiovascular disease, LDL-C= low-density lipoprotein cholesterol, HDL-C= high-density lipoprotein cholesterol, CAD=coronary artery disease, PCI= percutaneous coronary intervention. Pvalues from Kruskal–Wallis test (for continuous variables), Pearson chi-square tests (for categorical variables) and Fisher exact test (for categorical variables with small cell sizes).

**Supplementary Table 5**: 10-year MACE outcomes for baseline EPA + DHA quartiles.

|  | **EPA + DHA** | | | | | | | |  |  |
| --- | --- | --- | --- | --- | --- | --- | --- | --- | --- | --- |
|  | **Q1** | | **Q2** | | **Q3** | | **Q4** | | **Unadjusted**  **p-values** |  |
| **10 Year Outcomes** | **N=247**  Follow-up time  8.2±3.0 years | | **N=247**  Follow-up time  8.6±2.7 years | | **N=246**  Follow-up time  9.1±2.1 years | | **N=247**  Follow-up time  9.2±2.2 years | |  | **Adjusted**  **p-values** |
| **MACE** | 94 | 38.1% | 82 | 33.2% | 74 | 30.1% | 61 | 24.7% | 0.011 | 0.0009 |
| **Death** | 81 | 32.8% | 69 | 27.9% | 49 | 19.9% | 35 | 14.2% | <0.0001 | <0.0001 |
| **MI (>60 days)** | 5 | 2.0% | 10 | 4.1% | 7 | 2.8% | 10 | 4.1% | 0.65 | 0.75 |
| **Stroke** | 13 | 5.3% | 14 | 5.7% | 22 | 8.9% | 13 | 5.3% | 0.14 | 0.23 |
| **Heart Failure Admission** | 10 | 4.1% | 13 | 5.3% | 14 | 5.7% | 10 | 4.1% | 0.09 | 0.08 |

Note: EPA= eicosapentaenoic acid, DHA = docosahexaenoic acid, Q1=first quartile, Q2=second quartile, Q3=third quartile, Q4=forth quartile, MACE=major cardiovascular adverse events, MI=myocardial infarction. Cox proportional hazard regression used for both unadjusted and adjusted pvalues. Adjustment made for age, gender, hyperlipidemia, COPD, and severe CAD.

**Supplementary Figure 3:** Subgroup analyses; adjusted HR for 10-MACE by baseline EPA + DHA quartiles.

Note: EPA= eicosapentaenoic acid, DHA = docosahexaenoic acid, MACE=major cardiovascular adverse events, CAD=coronary artery disease, HF=heart failure, TG=triglyceride, HR=hazard ratio. Cox proportional hazard regression used with adjustment made for DHA, age, gender and significant comorbidities (hyperlipidemia, COPD, and severe CAD); variables removed from adjustment if used for subgroup stratification.

**Supplementary Table 6:** Baseline characteristics by baseline EPA / DHA ratio quartiles

|  | **EPA / DHA Ratio** | | | |  |
| --- | --- | --- | --- | --- | --- |
|  | **≤ 1** | | **>1** | |  |
|  | N=444 | | N=543 | | **Pvalue** |
| **Age, mean ± std** | 62.3 ± 12.1 | | 60.9 ± 12.2 | | 0.08 |
| **Male, No. (%)** | 239 | (53.8%) | 325 | (59.9%) | 0.06 |
| **Obese (BMI≥30), No. (%)** | 174 | (39.2%) | 234 | (43.1%) | 0.22 |
| **Smoking History** |  |  |  |  | 0.77 |
| Never | 335 | (75.4%) | 339 | (73.5%) |  |
| Former | 58 | (13.1%) | 75 | (13.8%) |  |
| Current | 51 | (11.5%) | 69 | (12.7%) |  |
| **Diabetic, No. (%)** | 149 | (33.6%) | 171 | (31.5%) | 0.49 |
| **Hx of Hypertension, No. (%)** | 274 | (61.7%) | 335 | (61.7%) | 1.0 |
| **Hx of Hyperlipidemia, No. (%)** | 207 | (46.6%) | 329 | (60.6%) | <0.0001 |
| **Hx of Heart Failure, No. (%)** | 35 | (7.9%) | 32 | (6.0%) | 0.22 |
| **Hx of AF, No. (%)** | 58 | (13.1%) | 50 | (9.2%) | 0.054 |
| **Hx of COPD, No. (%)** | 61 | (13.7%) | 52 | (9.6%) | 0.041 |
| **Hx. of Stroke, No. (%)** | 10 | (2.3%) | 10 | (1.8%) | 0.65 |
| **Hx of Depression, No. (%)** | 62 | (14.0%) | 89 | (16.4%) | 0.29 |
| **Family History of CAD, No. (%)** | 175 | (39.4%) | 264 | (48.6%) | 0.004 |
| **Prior Statin Use, No. (%)** | 121 | (27.3%) | 185 | (34.1%) | 0.02 |
| **Lipids** |  |  |  |  |  |
| Total Cholesterol, mg/dL (n=879) | 179.0 ± 40.7 | | 183.7 ± 43.4 | | 0.16 |
| LDL-C, mg/dL (n=828) | 105.9 ± 34.4 | | 107.1 ± 33.9 | | 0.61 |
| HDL-C, mg/dL (n=857) | 42.6 ± 13.5 | | 42.2 ± 13.2 | | 0.79 |
| Triglycerides, mg/dL (n=855) | 155.5 ± 99.3 | | 170.8 ± 120.1 | | 0.09 |
| **CAD** |  |  |  |  | 0.11 |
| No CAD | 191 | (43.0%) | 214 | (39.4%) |  |
| Mild/Moderate CAD | 83 | (18.7%) | 86 | (15.8%) |  |
| Severe CAD | 170 | (39.3%) | 243 | (44.8%) |  |
| **PCI Performed** | 82 | (18.5%) | 164 | (30.2%) | <0.0001 |

Note: EPA= eicosapentaenoic acid, Q1=first quartile, Q2=second quartile, Q3=third quartile, Q4=forth quartile, std=standard deviation, No.=number, BMI=body mass index, Hx=history, AF=atrial fibrillation, COPD= chronic obstructive pulmonary disease, CVD=cardiovascular disease, LDL-C= low-density lipoprotein cholesterol, HDL-C= high-density lipoprotein cholesterol, CAD=coronary artery disease, PCI= percutaneous coronary intervention. Pvalues from Kruskal–Wallis test (for continuous variables), Pearson chi-square tests (for categorical variables) and Fisher exact test (for categorical variables with small cell sizes).

**Supplementary Table 7**: 10-year MACE outcomes for baseline EPA /DHA

|  | **EPA / DHA Ratio** | | | |  |  |
| --- | --- | --- | --- | --- | --- | --- |
|  | **≤ 1** | | **>1** | | **Unadjusted**  **p-values** |  |
| **10 Year Outcomes** | **N=444**  Follow-up time  8.4±2.9 years | | **N=543**  Follow-up time  9.1 ± 2.2 years | |  | **Adjusted**  **p-values** |
| **MACE** | 163 | 36.7% | 148 | 27.3% | 0.001 | 0.013 |
| **Death** | 134 | 30.2% | 100 | 18.4% | <0.0001 | 0.0006 |
| **MI (>60 days)** | 10 | 2.3% | 22 | 4.1% | 0.10 | 0.10 |
| **Stroke** | 22 | 5.0% | 40 | 7.4% | 0.38 | 0.17 |
| **Heart Failure Admission** | 27 | 6.1% | 20 | 3.7% | 0.16 | 0.23 |

Note: EPA= eicosapentaenoic acid, DHA = docosahexaenoic aci, MACE=major cardiovascular adverse events, MI=myocardial infarction. Cox proportional hazard regression used for both unadjusted and adjusted pvalues. Adjustment made for age, gender, hyperlipidemia, COPD, prior statin use, family history of CAD and PCI.

**Supplementary Figure 4:** Subgroup analyses; adjusted HR for 10-MACE by baseline EPA /DHA (<1 vs ≥1).

Note: EPA= eicosapentaenoic acid, DHA = docosahexaenoic aci, MACE=major cardiovascular adverse events, MI=myocardial infarction. Cox proportional hazard regression used for both unadjusted and adjusted pvalues. Adjustment made for age, gender, hyperlipidemia, COPD, prior statin use, family history of CAD and PCI.

**Calculation of EPA and DHA mean absolute concentrations.**

We obtained absolute concentration values in a representative sample of specimens (n=100). These samples were sent to Boston Heart Diagnostics, Framingham, MA, to obtain the absolute concentration values of EPA and DHA. Using their results and doing a linear regression analysis with the LC-MS ASU values and the absolute concentration values, the following conversion equation was generated for EPA: EPA concentration (mcg/ml) = 1.642 + 0.8154 x ASU (x 10^-7^). For DHA, the concentration conversion equation was -12.56 + 2.60 x ASU (x 10^-7^). These two conversion equations resulted in an average EPA concentration of 20.42 ± 10.17 mcg/mL and an average DHA concentration of 52.14 ± 17.58 mcg/ml.
